# Insecticidal Mechanism of Botanical Crude Extracts and Their Silver Nanoliquids on *Phenacoccus solenopsis*

**DOI:** 10.3390/toxics11040305

**Published:** 2023-03-25

**Authors:** Mariappan Madasamy, Kitherian Sahayaraj, Samy M. Sayed, Laila A. Al-Shuraym, Parthas Selvaraj, Sayed-Ashraf El-Arnaouty, Koilraj Madasamy

**Affiliations:** 1 Crop Protection Research Centre (CPRC), Department of Zoology, St. Xavier’s College, Palayamkottai 627002, India; 2 Department of Science and Technology, University College-Ranyah, Taif University, P.O. Box 11099, Taif 21944, Saudi Arabia; 3 Department of Economic Entomology and Pesticides, Faculty of Agriculture, Cairo University, Giza 12613, Egypt; 4 Department of Biology, College of Science, Princess Nourah Bint Abdulrahman University, P.O. Box 84428, Riyadh 11671, Saudi Arabia; 5 Entomology Research Unit, Department of Zoology, St. Xavier’s College, Palayamkottai 627002, India

**Keywords:** botanical insecticides, AgNPs, enzymes, detoxification, macromolecules

## Abstract

In recent years, intensive studies have been carried out on the management of agricultural insect pests using botanical insecticides in order to decrease the associated environmental hazards. Many studies have tested and characterized the toxic action of plant extracts. Four plant extracts (*Justicia adhatoda*, *Ipomea carnea*, *Pongamia glabra*, and *Annona squamosa*) containing silver nanoparticles (AgNPs) were studied for their effects on *Phenacoccus solenopsis* Tinsley (Hemiptera: Pseudococcidae) using the leaf dip method. The effects were estimated based on assays of hydrolytic enzyme (amylase, protease, lipase, acid phosphatase, glycosidase, trehalase, phospholipase A2, and invertase) and detoxification enzyme (esterase and lactate dehydrogenase) levels; macromolecular content (total body protein, carbohydrate, and lipid); and protein profile. The results show that the total body of *P. solenopsis* contains trypsin, pepsin, invertase, lipase, and amylase, whereas *J. adathoda* and *I. carnea* aqueous extracts considerably decreased the protease and phospholipase A2 levels, and *A. squamosa* aqueous extract dramatically increased the trehalase level in a dose-dependent manner. The enzyme levels were dramatically decreased by *P. glabura*-AgNPs (invertase, protease, trehalase, lipase, and phospholipase A2); *I. carnea*-AgNPs (invertase, lipase, and phospholipase A2); *A. squamosa*-AgNPs (protease, phospholipase A2); and *J. adathoda*-AgNPs (protease, lipase, and acid phosphatase). Plant extracts and their AgNPs significantly reduced *P. solenopsis* esterase and lactate dehydrogenase levels in a dose-dependent manner. At higher concentrations (10%), all of the investigated plants and their AgNPs consistently decreased the total body carbohydrate, protein, and fat levels. It is clear that the plant extracts, either crude or together with AgNPs, may result in the insects having inadequate nutritional capacity, which will impact on all critical actions of the affected hydrolytic and detoxication enzymes.

## 1. Introduction

*Phenacoccus solenopsis* Tinsley (Hemiptera: Pseudococcidae), more commonly known as cotton mealybug, was initially identified as a nuisance in the USA. *P. solenopsis* has now spread to more than 43 nations and is mainly found in Pakistan, India, China, and Iran. Numerous other plant species, such as those of horticulture plants, field crops, and non-cultivated plants, can also become infected in addition to cotton plants [1,2]. A literature survey suggested that flexible, sustainable, multi-pest IPM strategies for pest management be implemented where botanicals are considered important components [3]. Many of the secondary metabolites that are present in plants help them defend themselves from diseases and herbivores. Secondary metabolites may function as precursors to physical defense mechanisms or have an antifeedant deterrent effect [3,4]. Many specialized herbivores and pathogens do not simply avoid the deterring or harmful effects of secondary metabolites but actually use them as either host recognition cues or nutrition [3]. A variety of biologically active chemicals that are produced by plants can function in both defense against herbivores in addition to influencing the growth and development of other species [3,4]. Insects are negatively impacted by secondary plant metabolites via acute toxicity [5], enzyme inhibition [6], and disruption of food ingestion or use [7]. Additionally, it has been suggested that plant-based formulations can affect physiological alterations via changing enzymes [6,8].

The first biological components to manifest throughout an insect’s development are proteins [9,10]. Proteins called enzymes catalyze a variety of biological processes [11]. Digestive enzymes are created and transported in varied proportions throughout the alimentary canal. Insects’ alimentary canals contain a wide range of digesting enzymes [11,12,13]. Among the various digestive enzymes, the activities of amylase, protease, lipase, invertase, acid phosphatase, and glycosidase play a crucial role in food digestion [14,15,16].

Insect herbivores can intensify their detoxifying processes against a specific plant toxin or xerobiosius [15]. According to studies, lactate dehydrogenase [10] and esterase [17] are thought to be significant detoxifying enzymes in insects. Due to its function in the detoxification and participation in the metabolism of a wide variety of exogenous and endogenous chemicals, esterase is crucial for insects [18]. Another significant glycolytic enzyme, lactate dehydrogenase (LDH), is essentially universally distributed throughout all tissues [19]. It is engaged in carbohydrate metabolism, which is utilized in toxicology and clinical chemistry to determine whether a patient has been exposed to phytochemical stress and to identify cell, tissue, and organ damage [20].

Knowledge about the role of the digestive enzymes occurring in the insect digestive tract is rather limited. This suggests that various metabolic pathways are activated by the ingestion of this prey, with the increasing of protein synthesis being of particular interest, as the control potential of *P. solenopsis* logarithmically increases in response to the protein content of their host [21]. Botanicals or their bioactive compounds are found to have an impact on many insects [22,23]. Because of its resistance to a variety of pesticides and the limits of other management measures when used as a single tactic, control of this pest requires a multidimensional approach [24,25].

Insecticidal activities of *Nicotiana glauca* Graham (Solanaceae), *Calotropis procera* Aiton (Apocynaceae) and *Guiera senegalensis* Adans (Combretaceae) [26], *C. procera*, *Azadirachta indica* Juss (Meliaceae), *Trachyspermum ammi* L. (Apiaceae), *Cardamine hirsute* L. (Brassicaceae), *Allium sativum* L. (Amaryllidaceae) and *Terminalia chebula* Retz (Combretaceae) [1], *Salvia rosmarinus* Spenn (Lamiaceae), *Cymbopogon citratus* Stapf (Poaceae), and *Eucalyptus melliodora* Cunn. (Myrtaceae) [2] were recorded against *P. solenopsis*. Additionally, against female *P. solenopsis*, the insecticidal efficacy of *Pelargonium graveolens* L’Hér. (Geraniaceae), *Thymus vulgaris* L. (Lamiaceae), and *C. citratus* essential oils (EOs) and silver nanoparticles (AgNPs) generated by employing the tested EOs were assessed [27]. Considering the lacuna in the available literature, the present investigations were conducted to determine the digestive and anti-toxicological physiological impacts of *Justicia adathoda* L. (Acanthaceae), *Pongamia glabra* L. (Fabaceae), *Annona squamosa* L. (Annonaceae), and *Ipomea carnea* Jacq. (Convolvulaceae) crude extracts and their AgNPs (0, 1.25, 2.5, 5.0, and 10%) and the commercial botanical pesticide vijayneem (0.03%) in *P. solenopsis*. Digestive physiological enzyme (amylase, protease, lipase, invertase, trehalase, glycosidase, acid phosphatase, phospholipase A2), anti-toxicological enzyme (esterase and lactate dehydrogenase), and macromolecule (total carbohydrates, total proteins, and total lipid contents) levels were recorded in *P. solenopsis* using the leaf dip application method.

## 2. Materials and Methods

### 2.1. Tested Plants

The botanicals were collected from Palayamkottai, Tirunelveli, Tamil Nadu, India with the locations as indicated in Table 1.

### 2.2. Preparation of Botanical Crude Extracts

In a 250 mL conical flask, 5 g of healthy, fresh leaves was dissected into small pieces. Distilled water (100 mL) was added to the flask, followed by boiling for 1 h in a water bath. After cooling, the decoctions were filtered using a Whatman No. 1 filter paper. The filtrates were collected in sterilized standard flasks of 50 mL and kept for use in experiments [28].

### 2.3. Preparation of Bio-Silver Nanoparticles

To prepare the sample, 99.9% pure silver nitrate (RM409-25G) from Hi-Media was employed. Distilled water (100 mL) was used to dissolve 17 mg of silver nitrate (10-3M AgNO_3_) [29]. In order to stimulate the creation of silver nanoparticles, each plant extract was supplied on a regular basis at intervals of 1 min. The final product, which ranged in color from pale yellow to reddish brown, was stored in a sterilized flask for further experiments.

### 2.4. Preparation of Commercial Vijayneem

Neem-based pesticides have been used by Indian farmers. The field recommended dose of 0.03% for vijayneem (Madras Fertilizers Limited, Chennai) was made (30 µL/100 mL water) and used for experiments.

### 2.5. Collection and Rearing of Pest

The life stages of *P. solenopsis* were obtained from cotton fields in Tirunelveli, Tamil Nadu, India’s Palayamkottai (longitude of E 77°66′57.73″ and latitude of N 8°73′70.75″). Under laboratory settings (89 cm width and 185 cm height) at room temperature of 29 ± 2 °C, 11 L: 13 D hours, and 70–80% R.H., *P. solenopsis* individuals were continuously reared on cotton plants and newly sprouted potato shoots in a plastic tray (24 cm width, 5 cm depth, and 30 cm height) for three generations [30]. For the experiment, the insects that emerged from the lab were likewise continually kept, and laboratory-emerged 1st, 2nd, and 3rd instar nymphs, as well as adults, of *P. solenopsis* were used.

### 2.6. Leaf Dip Method

Plastic containers were used to accomplish the leaf dip technique (5 cm height and 4 cm diameter). Four different aqueous extract concentrations of 1.25, 2.5, 5, and 10% were used. Healthy and fresh cotton leaves (KC-II) were cut into 2 cm square pieces and soaked in the test solutions for five minutes before being shade-dried for ten minutes [31]. The *P. solenopsis* adults and nymphs were fed on the shade-dried leaves. The experiment consisted of six replications. Teepol (50 µL) and distilled water (100 mL) were used as the control.

### 2.7. Enzyme Bioassay

Enzyme samples were prepared by the standard method [32]. After 144 h of the exposure period, living insects were placed on normal cotton leaves and maintained under laboratory conditions for a week. Then, the ten live animals were separately taken from experiments for each concentration (1.25, 2.5, 5, 10%, and control) of crude botanical extracts, bionanomaterials, and 0.03% of the commercial biopesticide vijayneem. The animals were decapitated in ice-cold insect ringer solution (100 mL of distilled water, 0.03 g of CaCl, 0.03 g of Na_2_CO_3_, 0.02 g of NaCl, and 0.025 g of KCl) and thoroughly washed in distilled water after being placed in the deep freezer (LG, Korea) for five minutes. The entire body of the *P. solenopsis* animal was weighed and then homogenized at 4 °C for 5 min in 1 mL of ice-cold phosphate buffer (pH 6.8), made from dissolving 1 g of NaH_2_PO_4_ and 1 g of disodium hydrogen orthophosphate in 100 mL of distilled water, and the volume then adjusted to 5 mL followed by thorough mixing. The supernatant from centrifugation of the homogenate at 5000 rpm for 15 min was utilized as an enzyme source (ES). Levels of the hydrolytic and detoxification enzymes amylase [33], protease [34], invertase [35], lipase [36], acid phosphatase [37], glycosidase [38], trehalase [39], phospholipase A_2_ [40], esterase [41], and lactate dehydrogenase [42] were quantified using standard procedures as described below.

#### 2.7.1. Qualitative Profiling of Digestive Enzymes

Ten live animals were taken and homogenized in 1 mL of phosphate buffer (pH 7.2) and then centrifuged at 3000 rpm for 30 min. The supernatants were collected and used for qualitative estimating analysis. To 100 µL of enzyme source, 100 µL of 2% starch was added, and the mixture was incubated in a water bath at 38 °C for 15 min. Then, a drop of this solution was placed in a porcelain tile, to which a drop of iodine was added. The appearance of blue color indicates the presence of amylase. All sample solutions turned blue, indicating the presence of amylase [35]. To 100 µL of enzyme source, 100 µL of 2% sucrose was added, and the mixture was incubated in a water bath at 38 °C for 30 min. Then, 200 µL Fehling’s solution A and B was added, followed by gentle heating in the water bath. All test solutions turned red, forming precipitate, indicating the presence of the enzyme invertase [35]. Two drops of olive oil or coconut oil and five drops of absolute alcohol were mixed, then gently heated, and an equal volume of distilled water was added followed by thorough mixing. To this, five drops of phenol red or bromophenol blue were added. To half of the above solution, 100 µL of enzyme source was added. The appearance of blue color indicates the presence of the enzyme lipase. All test solutions turned blue, indicating the presence of the enzyme invertase [35]. To 300 µL of enzyme source, 200 µL of 2% albumin was added, and the mixture incubated at 38 °C for 30 min. To this, 100 µL of 10% NaOH and 2 drops of 5% CuSO_4_ were added. The appearance of violet color indicates the presence of protease. All test solutions turned violet, indicating the presence of protease [34]. Then, 1% acetic acid was added to the reaction solution containing 500 µL alkaline casein and 500 µL enzyme extract that had been incubated at 26 °C for 6 h, where increased turbidity indicates tryptic activity [43]. For roughly six hours, the reaction solution of 500 µL casein (pH 2.0) and 500 µL enzyme extract was incubated at 26 °C and sodium acetate of 10% was added. The appearance of turbidity suggests the presence of pepsin [44].

#### 2.7.2. Quantitative Analysis of Hydrolytic Enzyme

##### Amylase

The Ishaaya test method [45] was applied. The reaction solution contained 0.2% (*w*/*v*) of soluble starch as the substrate, 0.25 mL of enzyme extract, and 1 mL of 10 mM borate buffer (pH 7.2). This was incubated in a water bath at 37 °C for 30 min. The same procedure was followed for the blanks except that the enzyme extract was not added. To stop the reaction, 0.4 mL of the 3,5-dinitrosalicyclic acid reagent was added, with heating at 100 °C for 5 min. Using a spectrophotometer set at 575 nm, the optical densities of the experimental and blank samples were measured and compared with a maltose standard. With g-maltose as the standard, the reducing sugars in weight (g-maltose) were expressed in moles per minute per mg.

##### Protease

Proteolytic activity was assessed [34] using Morihara and Tsuzuki’s technique and spectrometric analysis. A water bath was used to incubate the reaction solution comprising 1000 µL of 1% (*w*/*v*) casein solution and 500 µL of enzyme extract for 30 min at 35 °C. Then, 5 mL of 10% (*w*/*v*) trichloroacetic acid (TCA) was added to stop the reaction. The mixture was centrifugated at 3000 rpm for 10 min, and the supernatant was then collected. The method of Folin and Ciocalteu [46] was used to estimate the concentration of the digested protein. The supernatant (1 mL) and 500 µL of threefold-diluted Folin’s phenol reagent were added to 5 mL of Lowry’s reagent, carefully mixed, and incubated for 30 min at 26 °C. The optical density of the combination was estimated at 670 nm using a spectrophotometer in comparison with the blank, in which double-distilled water was used in place of the enzyme extract. The standard was tyrosine, and the protease activity was represented as µmoles of tyrosine liberated per min/mg of protein.

##### Invertase

Invertase activity of the soluble source was spectrophotometrically evaluated at 400 nm [35]. The ELICO INDIA SL 171 mini spectrophotometer was used for the analyses. For analysis, 25 mL of buffer solution was combined with 5 g of sample. A total of 500 µL of the sample solution was added to 5 mL of the substrate solution before being mixed together and incubated at 40 °C for 5 min. The reaction-terminating solution (500 µL) was added and thoroughly mixed exactly 20 min later. Prior to adding the sample solution for the blank, the reaction terminating solution was added. OD values at 400 nm were recorded. The invertase activity was measured in terms of the amount of phenol released per minute per mg of protein, with glucose serving as the reference.

##### Lipase

Cherry and Crandall’s [36] method was used to determine lipase activity. For this, 1 mL enzyme extract, 500 µL phosphate buffer, and 1 mL olive oil emulsion were used in the process. In the control group, distilled water was used instead of enzyme extract. Both tubes were shaking vigorously before being incubated for 12 h at 37 °C. Following the incubation period, the mixture was combined with 3 mL of absolute ethanol and two drops of phenolphthalein (2%) and then titrated against NaOH (0.05 N) solutions. The result was a pink color that appeared to be permanent. The titratable value of the experimental mixture was compared with that of the control. The following formula was used to determine lipase activity:

$$\mathrm{Lipase}\left(\mathrm{meq}/\min/g/\mathrm{sample}\right)=\frac{\mathrm{Consumed}\;\mathrm{volume}\;\mathrm{of}\;\mathrm{NaOH}\;\times \;\mathrm{Strength}\;\mathrm{of}\;\mathrm{alkali}}{\mathrm{Sample}\;\mathrm{Weight}\;\left(g\right)\times \mathrm{Time}\;\left(\min\right)}$$

##### Acid Phosphatase

The incubation mixture contained buffer (1.5 mL), substrate (1.5 mL), and the necessary amount of the enzyme source in a final volume of 3 mL [37]. For 15 min, each tube was incubated at 37 °C. The reaction was stopped by the addition of 1ml of Folin’s phenol reagent. After stopping the process, the enzyme was administered to the control tube. After centrifuging the contents, 1 mL of 15% sodium carbonate was added to the supernatant, and the mixture was then incubated for 10 min at 37 °C. Folin’s phenol reagent and sodium carbonate were used to treat the blank and standard, which contained aliquots of phenol. A UV spectrophotometer was used to read the color at 640 nm after treatment. The enzyme activity was measured in terms of moles of phenol emitted per minute per mg of protein, with P-nitrophenol serving as the reference.

##### Glycosidase

According to an established protocol [45], glycosidases were measured by spectrophotometrically monitoring the hydrolysis of 4-methylumbelliferyl- α-D-N-acetylneuraminic acid or nitrophenyl glycosides (for neuraminidase or all other glycosidases, respectively). For this, 400 µL of 0.1M phosphate buffer (pH 7.0) was added to 200 µL of extract. Then, 200 µL of 1% substrate was mixed, and a drop of toluene was added followed by incubation at 37 °C for 1 h. After incubation, 1.6 mL of DNS reagent was added, and the solution was then heated for 5 min at 100 °C in water bath and cooled in ice bath. Then, 1.6 mL of D. water was added with estimation at 550 nm. Glycosidic linkages, which are hydrolytic couplings that attach a carbohydrate to a peptide, a lipase, or another carbohydrate, were used to measure the enzyme activity. Only the terminal non-reducing monosaccharides of poly- or oligosaccharides are affected by exoglycosidases. P-Nitrophenol was employed as the reference substance, and the enzyme activity was reported as moles/minute/mg protein [47].

##### Trehalase

3,5-Dinitrosaliclic acid reagent was used to measure the free aldehyde groups of glucose generated after trehalose digestion in order to determine the amount of trehalase found in the enzyme source. The ideal enzyme reaction contains 0.2 mL of enzyme source, 1000 µL of 0.2 M acetate buffer (pH 3.5), and 100µL of 6% trehalose. Then, 800 µL of 3,5-dinitrosalicylic acid was added after incubation at 37 °C for 1 h. The solution was heated at 100 °C for 5 min and then rapidly cooled in an ice bath. At 550 nm, the optical density was measured using a spectrophotometer. One E unit equals 0.44 mg of glucose when glucose is directly reacted with dinitrosalicylic acid reagent under the same conditions as the enzyme assay. The enzyme activity was estimated as µmoles or µg glucose/per minute/mg enzyme reaction, and trehalase was used as a standard.

##### Phospholipase A_2_

The method of Santoro et al. [42] was used for determining phospholipase activity. For this, 1.5 mL of reaction solution (10 mM CaCl_2_, 100 mM NaCl, 7 mM Triton X-100, 98.8 M phenol red, and 0.265% egg lecithin, pH 7.6) was mixed with 15 µL of enzyme source. Immediately after homogenization, the solution was read at 558 nm. The amount of toxin (mg of protein/assay) producing a drop of 0.001 absorbance units/min under the conditions was used as the definition of 1 U of phospholipase A2 activity. Egg lecithin was employed as the reference, and the phospholipase activity was represented as moles/per minute/per mg.

#### 2.7.3. Detoxification Enzyme Esterase

The Van Asperen method [41] with minor modifications was used to determine the esterase activity. The enzyme extracts were 100 times diluted with 4 mM potassium phosphate buffer (pH = 8) and Triton X-100 (0.05%). After adding 500 µL of 0.5 mM naphthyl acetate in ethanol, 1 mL of the enzyme extract was incubated at 37 °C for 10 min. By adding 500 µL of dye solution (5% sodium lauryl sulfate; 1% diazoblue B salt = 2.5 V/U for 20 min), the process was stopped and color was produced. With a spectrophotometer, the absorbance was measured at 550 to 600 nm for -naphthol. The technique of Lowry et al. [46] was used to measure the concentration of protein using bovine serum albumin as the reference.

#### 2.7.4. Detoxification Enzyme Lactate Dehydrogenase

To the test and control tubes, which already contained 100 µL of each sample and 1000 µL of the buffer substrate, 200 µL of NAD solution and 200 µL of water, respectively, were added to standardize quantities. The sample reactions were arrested by adding 1000 µL of the colorant 2,4-dinitrophenylhydrazine to each tube. The incubation was carried out for 15 min at 37 °C. Then, 10 mL of NaOH (0.4 N) was added to each tube after the mixture had cooled to room temperature in order to make the solutions extremely alkaline and maximize hydrazine development. The color intensity in each tube was gauged at 440 nm 1 min after alkali had been added. The same process was applied to duplicate blanks that had standards. The chromogenicity of NADH2 generated in these tests is taken into account by adding the estimated amount of reduced Co enzymes to the standard. Multi-international units (MIU) per mg of protein per minute are used to express the enzyme activity [42]. One multi-international unit, with NAD as the reference, is the amount of enzyme needed to catalyze the conversion of 1 mg pyruvate to lactate or lactate to pyruvate/min/mL of the sample under the specified assay conditions.

### 2.8. Macromolecular Profile

The whole body of *P. solenopsis* was used to estimate the macromolecular contents, such as total protein, carbohydrate, and lipids. Ten healthy individuals were selected at random from the experimental and control groups, starved for 12 h, then put in a deep freezer for 5 min before being homogenized in phosphate buffer solution. The homogenate was employed for macromolecular studies. Total carbohydrate [48], total fat [49], and total protein [50] contents were represented in mg/g.

### 2.9. Statistical Analyses

Ten live animals were employed to prepare the macromolecular profiles and enzyme sources, as indicated in Section 2.7 and Section 2.8. The mean and standard deviation were computed after three replications of each test were completed. These data were also subjected to analysis of variance (ANOVA), and Tukey’s test was used to compare the means. The “P” values used to determine statistical significance were determined using the statistical package (SPSS V. 16.0). The *p* value was expressed at the 5% level.

## 3. Results

### 3.1. Qualitative Enzyme Profile

The data obtained from the qualitative analysis of the enzyme profile are summarized in Table 2. The results reveal the presence of amylase, protease, lipase, invertase, and maltase in *P. solenopsis*.

### 3.2. Crude Extracts on Hydrolytic Enzyme Quantitative Profile

**Figure 1:**
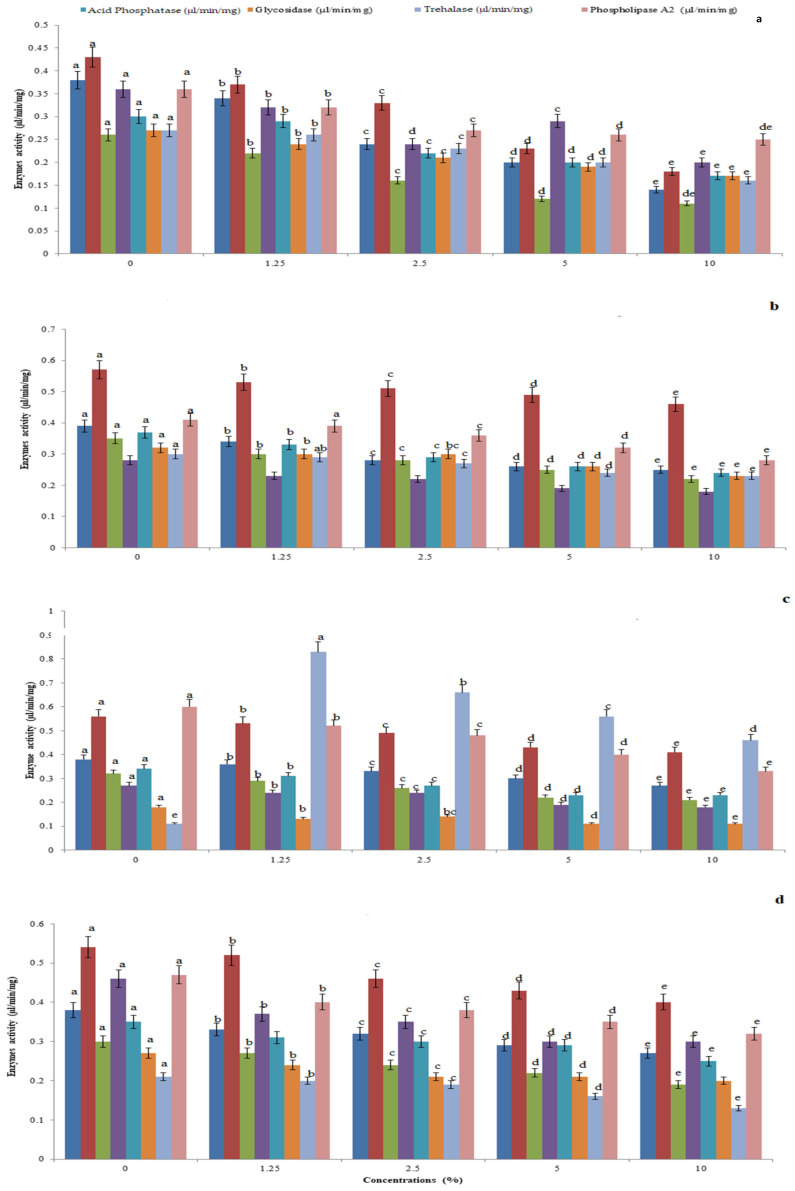
Whole insect body hydrolytic enzymes amylase, protease, invertase, lipase, acid phosphatase, glycosidase, trehalase, and phospholipase A_2_ levels of *P. solenopsis* exposed to *P. glabura* (**a**), *I. carnea* (**b**), *A. squamosa* (**c**), and *J. adathoda* (**d**) at different concentrations. Different letters (among the same concentration for each enzyme separately) above bars indicate significantly different means according to Tukey’s test (*p* < 0.05). Bars indicate the standard error (SE).

Invariably the amylase (F_4,10_ = 38.03, *p* = 0.002), protease (F_4,10_ = 31.128, *p* = 0.07), invertase (F_4,10_ = 24.236, *p* = 0.015), lipase (F_4,10_ = 5.214, *p* = 0.012), acid phosphatase (F_4,10_ = 55.241, *p* = 0.005), glycosidase (F_4,10_ = 67.25, *p* = 0.005), trehalase (F_4,10_ = 13.11, *p* < 0.001), and phospholipase A_2_ (F_4,10_ = 17.073, *p* = 0.005) activity was high in *P. glabura* (Figure 1a) and low in *I. carnea* ((F_4,10_ = 17.073, *p* = 0.005; F_4,10_ = 239.857, *p* = 0.038; F_4,10_ = 32.723, *p* < 0.01; F_4,10_ = 27.173, *p* = 0.01; F_4,10_= 75.531, *p* = 0.05; F_4,10_ = 17.985, *p* = 0.05; F_4,10_ = 38.045, *p* = 0.05; and F_4,10_ = 23.915, *p* < 0.05) for amylase, protease, invertase, lipase, acid phosphatase, glycosidase, trehalase, and phospholipase A_2_, respectively) (Figure 1b). Both *A. squamosa* (Figure 1c) and *J. adathoda* (Figure 1d) showed moderate impacts.

### 3.3. Effect of Bionanoliquid on Hydrolytic Enzyme Quantitative Profile

**Figure 2:**
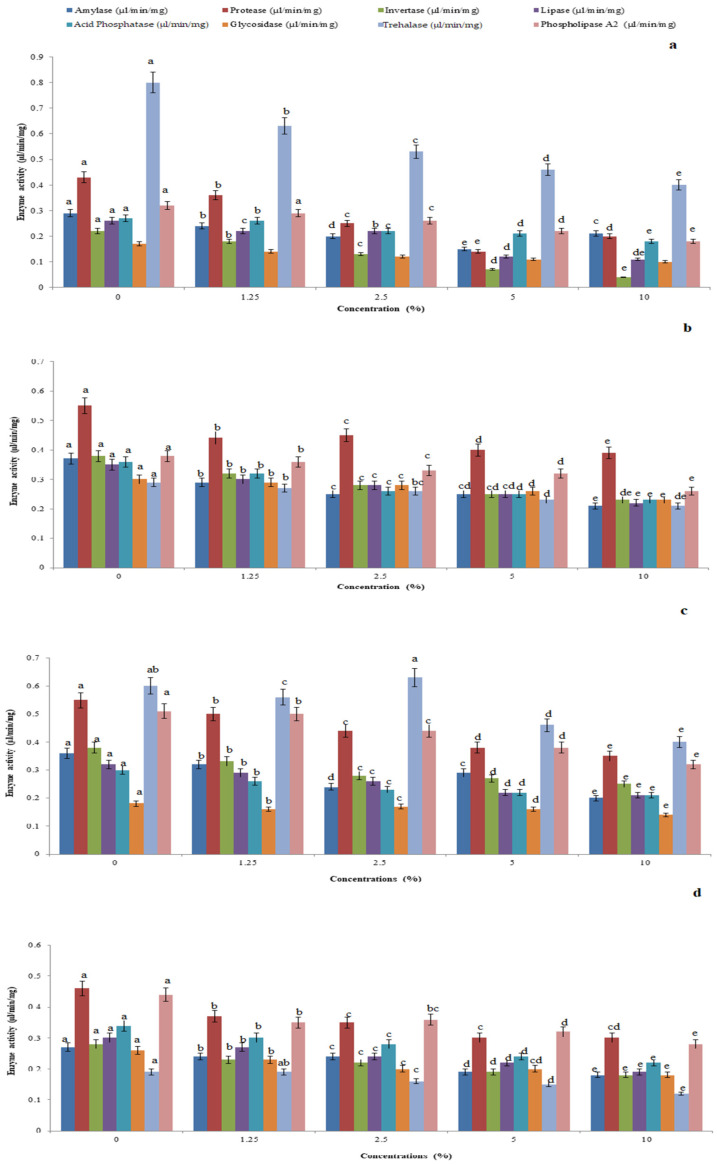
Whole insect body hydrolytic enzymes amylase, protease, invertase, lipase, acid phosphatase, glycosidase, trehalase, and phospholipase A_2_ levels of *P. solenopsis* exposed to *P. glabura*-AgNPs (**a**), *I. carnea-* AgNPs (**b**), *A. squamosa-*AgNPs (**c**), and *J. adathoda-*AgNPs (**d**) at different concentrations. Different letters (among the same concentration for each enzyme separately) above bars indicate significantly different means according to Tukey’s test (*p* < 0.05). Bars indicate the standard error (SE).

*P. glabra*-AgNP-treated *P. solenopsis* showed higher levels of amylase (F_4,10_ = 1.285, *p* = 0.219), protease (F_4,10_ = 37.551, *p* = 0.052), invertase (F_4,10_ = 11.211, *p* = 0.001), lipase (F_4,10_ = 24.236, *p* = 0.0015), acid phosphatase (F_4,10_ = 45.429, *p* = 0.005), glycosidase (F_4,10_ = 126.167, *p* = 0.005), trehalase (F_4,10_ = 36.667, *p* = 0.005), and phospholipase A_2_ (F_4,10_ = 46.339, *p* = 0.005) level (Figure 2a). As observed for the crude extracts, *I. carnea*-AgNP-treated *P. solenopsis* showed the least impact ((F_4,10_ = 15.591, *p* = 0.004; F_4,10_ = 14.435, *p* = 0.059; F_4,10_ = 30.925, *p* = 0.09; F_4,10_ = 32.724, *p* = 0.01; F_4,10_ = 182.286, *p* = 0.05; F_4,10_ = 11.788, *p* = 0.01; F_4,10_ = 22.05, *p* = 0.05; and F_4,10_ = 23.538, *p* = 0.05) for amylase, protease, invertase, lipase, acid phosphatase_,_ glycosidase, trehalase, and phospholipase A_2_, respectively) (Figure 2b). AS-AgNPs (Figure 2c) and JA-AgNPs (Figure 2d) caused moderate impacts on *P. solenopsis*.

### 3.4. Hydrolytic and Detoxification Enzyme Profile of Vijayneem

**Figure 3:**
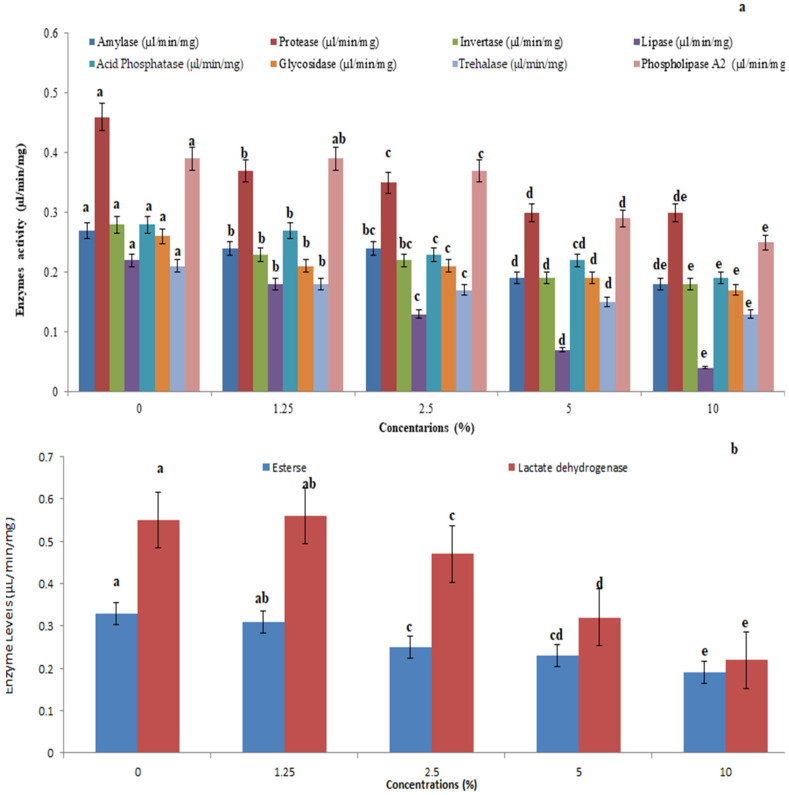
Whole insect body hydrolytic enzyme levels of amylase, protease, invertase, lipase, acid phosphatase, glycosidase, trehalase, and phospholipase A_2_ (**a**) and levels of detoxification enzymes esterase and lactate dehydrogenase (**b**) in insect *P. solenopsis* exposed to vijayneem at different concentrations. Different letters (among the same concentration for each enzyme separately) above bars indicate significantly different means according to Tukey’s test (*p* < 0.05). Bars indicate the standard error (SE).

Vijayneem-treated *P. solenopsis* showed higher levels of amylase (F_4,10_ = 12.073, *p* = 0.003), protease (F_4,10_ = 10.991, *p* = 0.043), invertase (F_4,10_ = 30.37, *p* < 0.001), lipase (F_4,10_ = 11.211, *p* = 0.001), acid phosphatase (F_4,10_ = 25.214, *p* = 0.005), glycosidase (F_4,10_ = 50.389, *p* = 0.005), trehalase (F_4,10_ = 16.00, *p* = 0.005), phospholipase (F_4,10_ = 141.167, *p* = 0.005) (Figure 3a), and esterase (F_4,10_ = 271.00, *p* = 0.005) followed by lactate dehydrogenase (F_4,10_ = 54.427, *p* = 0.005) (Figure 3b).

### 3.5. Detoxification Enzyme Profile for Plant Crude Extracts and Their Nanoliquid

**Figure 4:**
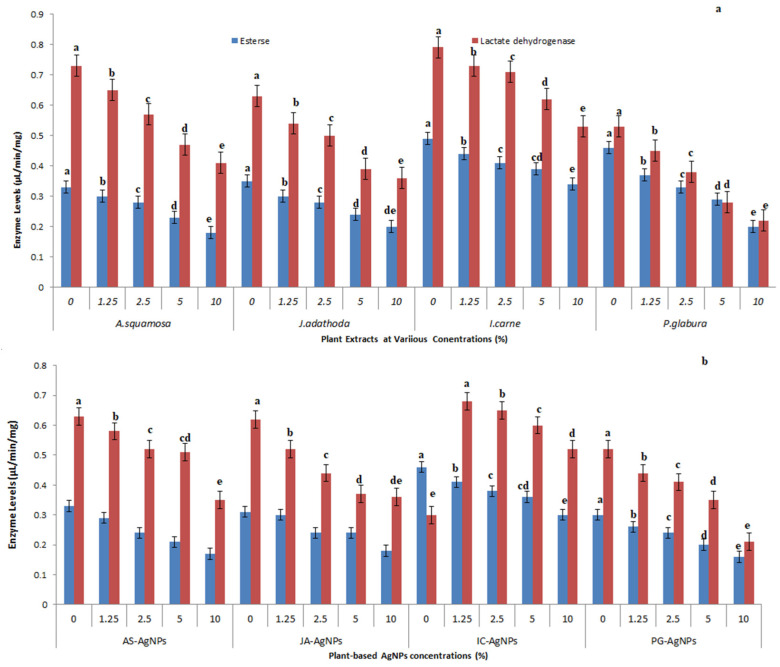
Whole insect body detoxification enzyme esterase and lactate dehydrogenase levels of insect *P. solenopsis* exposed to *P. glabura*, *I. carnea*, *A. squamosa*, and *J. adathoda* (**a**) and *AS-*AgNPs, *JA-*AgNPs, *IC-*AgNPs, and *PG*-AgNPs (**b**) at different concentrations. Different letters (among the same concentration for each enzyme separately) above bars indicate significantly different means according to Tukey’s test (*p* < 0.05). Bars indicate the standard error (SE).

The esterase and lactate dehydrogenase activities were highest in *P. solenopsis* treated with *P. glabura* crude extract (F_4,10_ = 131.063, *p* = 0.005 and F_4,10_ = 58.285, *p* = 0.005 for esterase and lactate dehydrogenase, respectively) and the lowest enzyme levels were observed for *I. carnea* crude extract treatment (F_4,10_ = 53.796, *p* =0.005 and F_4,10_ = 42.327, *p* = 0.005 for esterase and lactate dehydrogenase, respectively) (Figure 4a). A similar observation was also made for *P. glabra*-AgNPs (F_4,10_ = 36.513, *p* = 0.005 and F_4,10_ = 58.285, *p* = 0.005 for esterase and lactate dehydrogenase, respectively) and *I. carnea*-AgNPs (F_4,10_ = 94.00, *p* < 0.05 and F_4,10_ = 18.774, *p* < 0.05 for esterase and lactate dehydrogenase, respectively) in *P. solenopsis* treated with AS-AgNPs and JA-AgNPs (Figure 4b).

### 3.6. Macromolecular Profile

**Figure 5:**
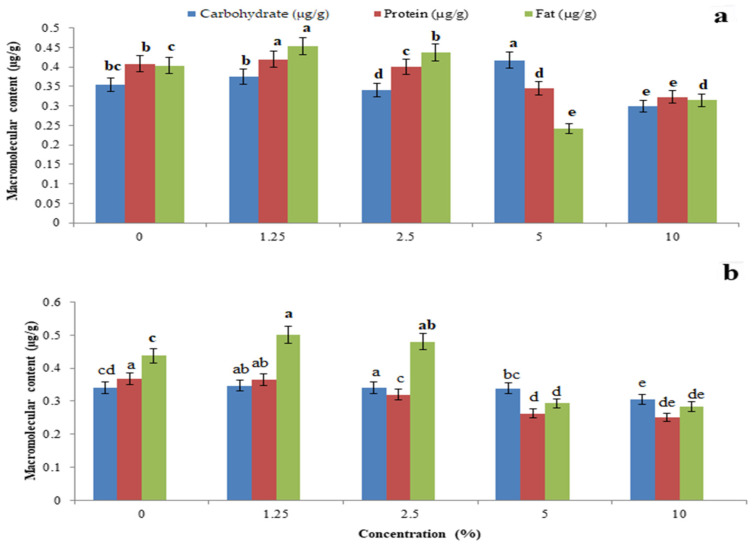
Whole-body carbohydrate, protein, and fat (µg/g) of *P. solenopsis* exposed to different concentrations of *P. galabura* plant extract (**a**) and *P. galabura-*AgNPs (**b**). Different letters (among the same concentration for each enzyme separately) above bars indicate significantly different means according to Tukey’s test (*p* < 0.05). Bars indicate the standard error (SE).

**Figure 6:**
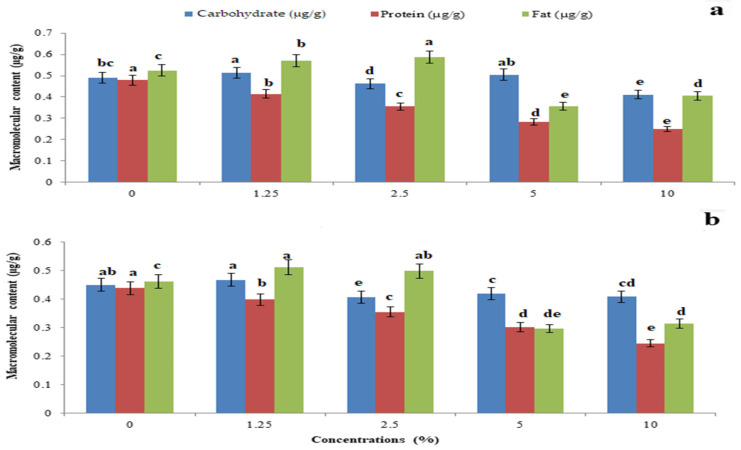
Whole-body carbohydrate, protein, and fat (µg/g) of *P. solenopsis* exposed to different concentrations of *I. carnea* (**a**) and *I. carnea-*AgNPs (**b**). Different letters (among the same concentration for each enzyme separately) above bars indicate significantly different means according to Tukey’s test (*p* < 0.05). Bars indicate the standard error (SE).

**Figure 7:**
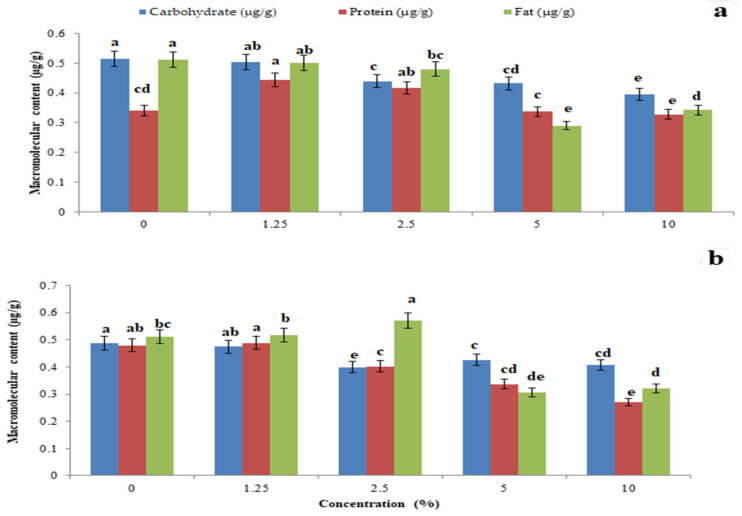
Whole-body carbohydrate, protein, and fat (µg/g) of *P. solenopsis* exposed to different concentrations of *A. squamosa* (**a**) and *A. squamosa-*AgNPs (**b**). Different letters (among the same concentration for each enzyme separately) above bars indicate significantly different means according to Tukey’s test (*p* < 0.05). Bars indicate the standard error (SE).

**Figure 8:**
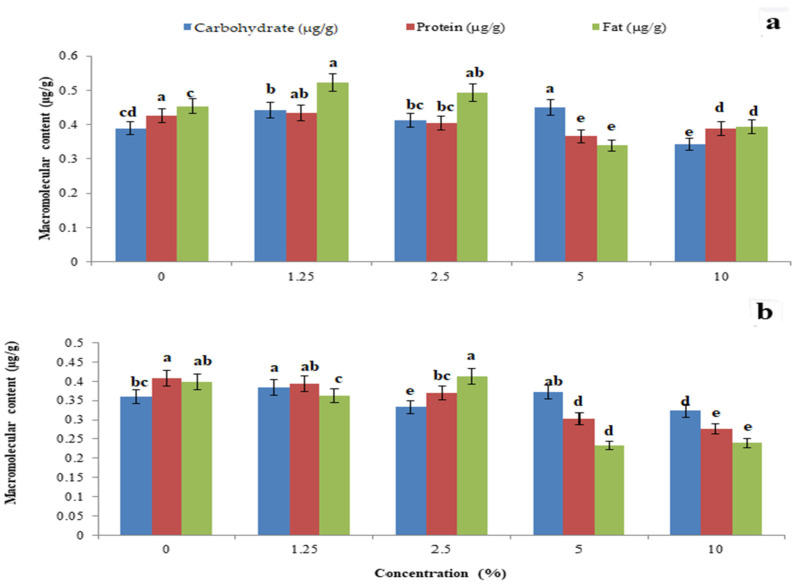
Whole-body carbohydrate, protein, and fat (µg/g) of *P. solenopsis* exposed to different concentrations of *J. adathoda* (**a**) and *J. adathoda-*AgNPs (**b**). Different letters (among the same concentration for each enzyme separately) above bars indicate significantly different means according to Tukey’s test (*p* < 0.05). Bars indicate the standard error (SE).

**Figure 9:**
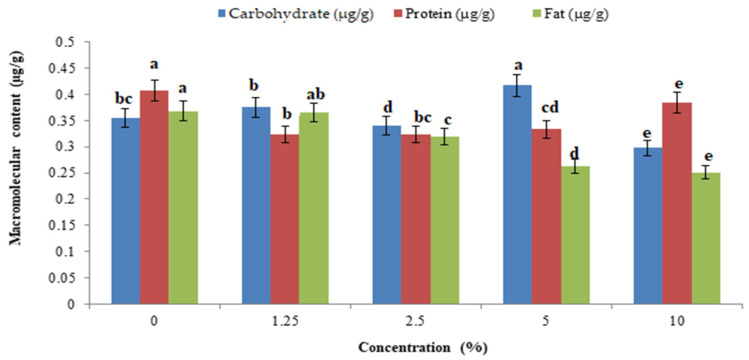
Whole-body carbohydrate, protein, and fat (µg/g) of *P. solenopsis* exposed to different concentrations of vijayneem. Different letters (among the same concentration for each enzyme separately) above bars indicate significantly different means according to Tukey’s test (*p* < 0.05). Bars indicate the standard error (SE).

The total body carbohydrate content was significantly reduced by *P. glabra* plant extract (F_4,10_ = 22.485, *p* = 0.001) (Figure 5a) and *p. glabra*-AgNPs (F_4,10_ = 1.797, *p* = 0.006) (Figure 5b) than *I. carnea* (F_4,10_ = 14.765, *p* = 0.005) (Figure 6a) and *I. carnea*-AgNPs (F_4,10_ = 3.554, *p* = 0.001) (Figure 6b), compared with *A. squamosa* (Figure 7a,b), *J. adathoda* (Figure 8a,b), and also vijayneem (F_4,10_ = 22.485; *p* < 0.001) (Figure 9). Similar results were also observed for total body protein (F_4,10_ = 7.68, *p* < 0.001; F_4,10_ = 35.2, *p* = 0.03; and F_4,10_ = 6.43, *p* < 0.01 for *P. glabra*, *I. carnea* crude extract, and vijayneem, respectively) and lipid content (F_4,10_ = 40.34, *p* < 0.001; F_4,10_ = 176.47, *p* < 0.01; and F_4,10_ = 6.912, *p* = 0.08 for *P. glabra*, *I. carnea* crude extract, and vijayneem, respectively).

## 4. Discussion

The plant extracts and their AgNPs showed insecticidal efficacy on *P. solenopsis*. Moreover, *P. solenopsis* nymphal period, adult longevity, fertility, and hatchability were all decreased by nanoparticles and plant extracts (unpublished data). Thus, this study aimed to investigate the biochemical mechanisms of crude extracts of the same plants containing AgNPs against mealybug. Hydrolytic enzymes convert complicated dietary components into the micromolecules needed to provide energy and metabolites in the bodies of insects. In the various gut parts or whole-body compartments, hydrolytic enzymes involved in primary digestion—cleaving polymers such as protein and starch—secondary digestion acting on oligomers such as polypeptides and dextran—and final digestion hydrolyzing dimers such as dipeptides and disaccharides—were measured. Feeding is essential for promoting enzyme activity [50], and it can affect the control of digestive enzyme synthesis at either the transcriptional or translational level. The alimentary canal is where many of the digestive enzymes, such as lipase, carboxylase, amylase, invertase, protease, and maltase, are generated and secreted in most insects [7,15].

The results revealed that *P. solenopsis* digestive and detoxication enzymes are strongly influenced by the tested botanical extracts and their bio-AgNPs as well as vijayneem. Invariably, enzyme activity is directly proportional to the concentrations of crude plant extracts similarly to other bionanoliquid treatments. The reduced levels of digestive enzymes at higher concentrations of the tested plant extracts and their silver bionanoliquid indicates reduced phosphorous liberation for energy metabolism as well as slower metabolism and a lower rate of metabolite transportation, which may be caused by the direct impacts of botanical extracts on enzyme regulation [51].

Among the tested plants, *P. glabura* crude extracts with AgNPs significantly reduced levels of digestive enzymes (amylase, protease, lipase, invertase, trehalase, glycosidase, acid phosphatase, and phospholipase A2), anti-toxicological enzymes (lactate, esterase, and dehydrogenase), and total body macromolecules (total protein, carbohydrate, and lipid content) due to the impact of antifeedant compounds glabrachromene-I [52], 3′-methoxy-pongapin [53], 4′–methoxy furano (2′,3′: 7,8)/–flavone [54], diglycoside, 4-O-methyl-genistein 7-O-d-rutinoside, isoflavanoid, 2,5-dimethoxy-genistein 7-O-d-apiofuranosyl-(156)-O-d-glucopyranoside; rotenoid, 12a-hydroxy-α-toxicarol; vecinin-2 (1), rutin, vitexin, kaempferol 3-O-d-rutinoside, isoquercitrin, 11,12a-dihydroxy-munduserone, kaempferol 3-O-d-glucopyranoside, kaempferol, and quercetin found in *P. glabra* leaves, as shown in many investigations [55,56,57]. *P. glabura* has shown insecticidal activity against *Nilaparvata lugens* Stål. (Homoptera: Delphacidae) [56], a wide range of insect pests [57], *Tribolium castaneum* Herbst (Coleoptera: Tenebrionidae [58], and *Leucinodes orbonalis* Guenee (Lepidoptera: Pyrallidae) [59].

As seen in this work, *P. glabra* leaf extracts effectively decreased the invertase, amylase, and protease activities in *Euproctis fratema* Moore (Lepidoptera: Erebidae) fifth instar larvae [60]. The antifeedant compound’s inhibition of protease activity may have a direct effect on a physiological system that affects protease activity. Many plant defense compounds inhibit digestive enzymes such as amylase [61], proteases [62], and lipase [51]. Low activity of whole-body proteases indicates great de-utilization of exogenous proteins in more physiological activities for development. The protease enzyme was responsible for the accumulation of major digestion, and the highest quantities are exhibited in response to a high concentration of nanoparticles, vijayneem, and then crude extracts. Protease was reduced by treatment with the nanoparticles in *Helicoverpa armigera* [63], and crude extracts of *Hyphantria cunea* [64] as observed in *P. solenopsis*. Vijayneem blocks the secretion of invertase, which may have importance for the utilization and digestion of sucrose in insects [65].

Acid phosphatase enzymes are secreted in the digestive track of insects and have important roles in the utilization and digestion of sucrose and starch, respectively. The reduction observed in acid phosphatase activity is similar to results reported earlier [31]. In treated flies, the digestive enzyme activity may have been inhibited by the reduced availability of these substrates [20]. It was shown that the rupture of epithelial cells, which are the sites for enzyme secretion, may be the cause of the reduction in alkaline phosphatase and amylase activities as a result of *Hemionitis arifolia* L. (Pteridaceae) and *P. glabra* treatments [14].

Insect herbivores can boost their detoxifying activities against a specific plant poison/toxin after ingesting the same components for a long or short period of time. According to studies, the second phase of enzyme detoxification involves the activities of glutathione P-transferase, glutathione S-transferase, alanine aminotransferase (ALAT), transoxidase hydrolases, glutathione transferase, and cytochrome P-450 [66]. Generally, detoxification enzymes include cytochrome P450 monooxygenases (P450s), glutathione S-transferases (GSTs), carboxylesterases (CarEs), and adenosine triphosphate (ATP) binding cassette (ABC) transporters [67]. Additionally, Upadhyay [68] reported, for the first time, investigation of those acid and alkaline phosphatases as enzymes important in detoxification. A number of predators were also found to be affected by plant allelochemicals on GST activity, in addition to herbivores [18]. The levels of detoxification enzymes decrease while the concentrations of nanoparticles decrease. This indicates that the animal attempts to respond to decrease their effects based on bioactive principle levels. The lactate dehydrogenase activity is high in nanoparticles and plant extracts compared with vijayneem. Otherwise, the activity of the lactate dehydrogenase enzyme decreases due to chemical stress [20].

Whole-body total carbohydrate, protein, and fat content was significantly decreased by Pg-AgNPs, vijayneem, or *P. galabura* plant extract-treated cotton leaf-fed *P. solenopsis*. As a result of ingesting *Azadirachta indica* oil and vijayneem-treated leaves, *Spodoptera litura* Fab. produced more lipid (4.0, 10.4%), protein (4.7, 7.7%), and carbohydrate (27.0, 18.0%) [69]. *P. galabura* components such as pungamin, glabrin, karajin, karanjae, and pongaglabrone chemicals are responsible for the reduction of carbohydrate content in *P. solenopsis*.

Lipids are important macromolecules that help increase the utilization of energy from feed. The total lipid content of *P. solenopsis* was highly increased by plant extracts, or vijayneem, and also by the different concentrations of AgNPs. However, the lipid content was reduced by AgNP-treated cotton leaf-fed *P. solenopsis,* considering the nanoparticles of plant leaf extract and silver react with the reduced lipid content of *P. solenopsis*. The reason for decreasing total lipid content may be in terms of their conversion to proteins as a substitute for the decrease in protein content or the production of supplementary energy [70]. The gut wall has a significant capacity to rapidly utilize these dietary lipids, which it incorporates into phospholipids [71]. Moreover, apart from the direct lethal effect induced by xenobiotics, sublethal effects likely occur [72], including effects on insect biology, physiology, behavior, and demographic parameters, such as survival rate, developmental rate, longevity, fecundity, fertility, mating behavior, food searching, and oviposition [73].

## 5. Conclusions

According to the findings, the qualitative enzyme profiling shows that the *P. solenopsis* whole body contains amylase, invertase, lipase, protease, trypsin, and pepsin. *P. glabura*-AgNPs and *P. glabura* aqueous extract both dramatically decreased hydrolytic enzymes (amylase, protease, invertase, acid phosphatase, glycosidase, and phospholipase A2 levels) in *P. solenopsis* in a dose-dependent manner. The levels of *P. solenopsis* protease, lipase, acid phosphatase, glycosidase, trehalase, and phospholipase A2 were likewise markedly lowered by the commercial neem-based product vijayneem in a dose-dependent manner. In a dose-dependent way, plant extracts and their AgNPs drastically decreased *P. solenopsis* esterase and lactate dehydrogenase. All of the examined plants and their AgNPs consistently reduced the total body’s carbohydrate, protein, and fat contents at greater concentrations (10%). It is evident that the insect’s poor nutritional capacity may be the root of the effects of the plant’s crude extracts, either alone or with AgNPs, which will simultaneously affect all subsequent crucial actions of hydrolytic and detoxication enzymes. These results demonstrate the crucial role for these compounds in the fight against agricultural pests and could be employed to boost their effectiveness and quality in the future.

## Figures and Tables

**Table 1:** Botanicals and their locations used in the current study.

Plant Species	Family	Longitude (E)	Latitude (N)
*Justicia adhatoda*	Acanthaceae	77°73′81.22″	08°71′80.23″
*Ipomea carnea*	Convolvulaceae	77°68′75.15″	08°73′05.34″
*Pongamia glabra*	Fabaceae	77°74′01.66″	08°71′74.48″
*Annona squamosa*	Annonaceae	77°66′57.73″	08°73′70.75″

Whole collected plants were washed three times with distilled water for removing debris and dust.

**Table 2:** Qualitative enzyme analysis of *P. solenopsis’s* whole body.

Name of the Qualitative Enzyme	Sample of Whole-Body Insect
Amylase	+
Invertase	++
Lipase	++
Protease	++
Trypsin	+
Pepsin	+

Less (+), moderate (++) enzyme activity of *P. solenopsis*.

## Data Availability

All data belonging to the current research are available from the corresponding authors on reasonable request.
